# How do I steer this thing? Using dendritic cell targeted vaccination to more effectively guide the antitumor immune response with combination immunotherapy

**DOI:** 10.1186/s40425-016-0135-z

**Published:** 2016-06-21

**Authors:** Stefanie N. Linch, William L. Redmond

**Affiliations:** Robert W. Franz Cancer Research Center, Earle A. Chiles Research Institute, Providence Portland Medical Center, 4805 NE Glisan St. 2N35, Portland, OR 97213 USA; Department of Molecular Microbiology and Immunology, School of Medicine, Oregon Health and Science University, Portland, OR 97239 USA

**Keywords:** Immunotherapy, Cytotoxic CD8 T cell, OX40, CTLA-4, Checkpoint blockade, Co-stimulation, Dendritic cell, Vaccine, Anergy, Tolerance

## Abstract

Mounting an immune response sufficient to eradicate a tumor is the goal of modern immunotherapy. Single agent therapies with checkpoint inhibitors or costimulatory molecule agonists are effective only for a small portion of all treated patients. Combined therapy, e.g., CTLA-4 and PD-1 checkpoint blockade, is a more effective treatment modality, but in preclinical studies OX40 agonism with CTLA-4 blockade using monoclonal antibodies (aOX40/aCTLA-4) failed to induce tumor regression of larger, more established tumors. We hypothesized that administration of a vaccine with a tumor-associated antigen targeted to the appropriate antigen presenting cell could make combined aOX40/aCTLA-4 therapy more effective. We administered an antibody-based vaccine targeting HER2 to the DEC-205 endocytic receptor on cross-presenting dendritic cells (anti-DEC-205/HER2; aDEC-205/HER2) and a potent adjuvant (poly (I:C)) to assist with maturation, along with aOX40/aCTLA-4 therapy. This therapy induced complete regression of established tumors and a pronounced infiltration of effector CD8 and CD4 T cells, with no effect on regulatory T cell infiltration compared to aOX40/aCTLA-4 alone. To be maximally effective, this therapy required expression of both OX40 and CTLA-4 on CD8 T cells. These data indicate that vaccination targeting cross-presenting dendritic cells with a tumor-associated antigen is a highly effective immunization strategy that can overcome some of the limitations of current systemic immunotherapeutic approaches that lack defined tumor-directed antigenic targets.

## Background

Immunotherapy is quickly garnering attention and enthusiasm as some patients with metastatic disease have achieved long-term remission. However, combinations of immunotherapies and/or targeted therapies will be needed to achieve complete tumor regression for a larger portion of patients. Our lab has been investigating the efficacy of OX40 agonism in combination with CTLA-4 blockade. OX40 is costimulatory molecule expressed by both CD4 and CD8 T cells following T cell receptor (TCR) ligation [1]. Preclinical data demonstrate that treatment with agonist anti-OX40 monoclonal antibodies (aOX40) induced tumor regression by boosting effector CD8 and CD4 T cell expansion and function [2–6]. Another successful approach is the blockade of a co-inhibitory molecule, CTLA-4, which limits an active immune response. Our previous research has demonstrated that combination aOX40/aCTLA-4 therapy significantly improved survival in preclinical models [7]. Surprisingly, this therapy also induced a profound Th2 bias in CD4 T cells. It is known that TCR-mediated recognition of low-affinity antigens can promote a Th2 bias, which limits an effective antitumor immune response, and that promoting a Th1 bias results in more favorable outcomes for patients [8–13]. In order to circumvent a Th2 bias and promote a more robust Th1 response, we opted to augment a CD8 T cell response directly via DEC205 expressing cross-presenting dendritic cells (DCs) [14]. It was previously demonstrated that mice defective in cross-presentation have impaired tumor rejection and that in cancer, DC function is frequently impaired [15, 16]. We hypothesized that vaccination targeting a tumor-associated antigen toward cross-presenting dendritic cells (aDEC-205/HER2 with poly (I: C)) combined with aOX40/aCTLA-4 immunotherapy would promote a robust effector CD8 T cell response capable of clearing established tumors.

## Main text

**Fig. 1:**
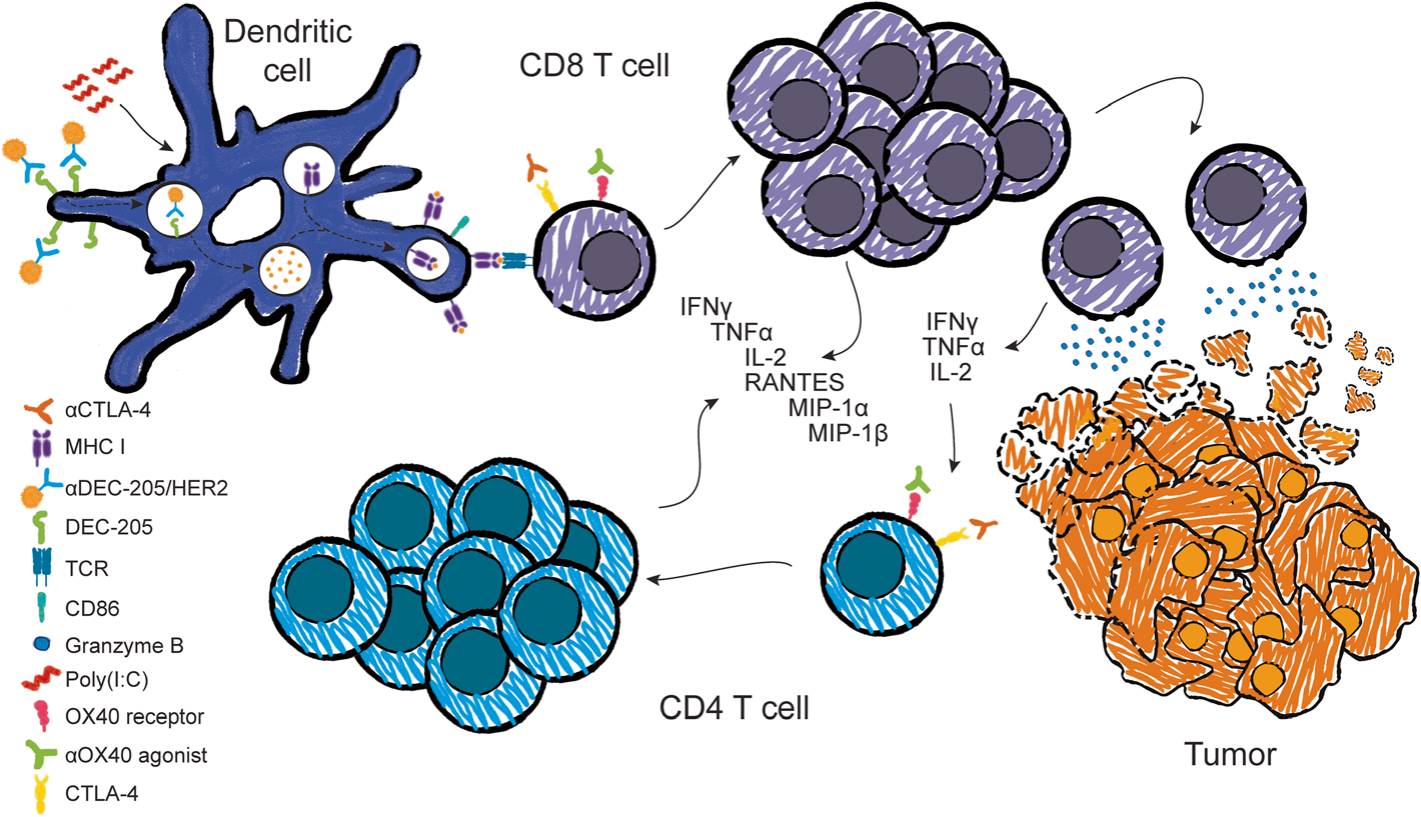
Vaccination using aDEC-205/HER2 combined with adjuvant poly (I:C) induces dendritic cell maturation and costimulatory molecule expression, thereby resulting in more efficient antigen presentation to CD8 T cells. Activation of CD8 T cells via the T cell receptor (TCR) and OX40 using an OX40 agonist induces robust CTL activation, while CTLA-4 blockade releases the brakes on the activated CTL. Effector CD8 T cells can now traffic into the tumor, where they accumulate and induce cancer cell death using cytolytic granule proteins. T cell activation and cancer cell death leads to increased cytokine (IFN-γ, TNF-α, IL-2) and chemokine (CCL3/MIP-1α; CCL4/MIP-1β; CCL5/RANTES) production leading to additional recruitment of effector T cells. OX40 agonism and CTLA-4 blockade also lead to CD4 T cell activation and expansion. Together, this robust T cell response results in tumor eradication and improved long-term survival

To elaborate on our previous studies, we tested the effect of combination aOX40/aCTLA-4 therapy on antigen-specific T cell expansion and the kinetics of this response. Combination aOX40/aCTLA-4 therapy significantly increased the frequency, function, and persistence of antigen-specific CD8 T cells in the periphery over time. To determine whether this was a direct or indirect effect on CD8 T cells, we used OX40-deficient and human CTLA-4 knock-in transgenic mice. OX40^-/-^ OT-I cells had a significantly reduced ability to proliferate, differentiate into effector cells, and produce inflammatory cytokines following combination therapy, indicating the requirement for OX40. To determine whether CTLA-4 expression on CD8 T cells was required for the efficacy of combination therapy, we used transgenic mice in which the extracellular portion of the mouse CTLA-4 receptor is swapped with the human version (huCTLA-4 mice), rendering them unresponsive to anti-mouse CTLA-4 antagonism [17]. Surprisingly, CTLA-4 expression on CD8 T cells was required to induce maximal expansion and function of this population following combined aOX40/aCTLA-4 treatment. Furthermore, CD4 T cells were required to induce a potent CD8 T cell response. A key observation we made in our previous study was that aOX40/aCTLA-4 therapy was not sufficient to improve survival of mice with larger, more established tumors. Notably, when aDEC-205/HER2 vaccination was combined with aOX40/aCTLA-4, we observed regression of established tumors (100-150 mm^2^). This corresponded with a significant increase in inflammatory cytokine and chemokine production by CD4 and CD8 T cells, and a notable decrease in Th2 cytokines from CD4 T cells, which we had observed previously. The triple combination induced profound CD8 and CD4 effector T cell infiltration in the tumor. It is known that T cell anergy is a major obstacle to effective antitumor immunity. To investigate whether this triple combination could overcome T cell anergy, we combined a mouse model of anergy using POET-1 (probasin ovalbumin expressing transgenic-1) combined with a spontaneous prostate cancer model – TRAMP (transgenic adenocarcinoma of the mouse prostate) transgenic mice [18, 19]. POET-1 mice express membrane-bound ovalbumin (mOVA) in the prostate driven by the rat probasin promoter. Thus, POET-1 x TRAMP (TRAMP-OVA) mice express mOVA as a self/tumor-associated antigen that renders ovalbumin-specific CD8 T cells anergic. Combined aOX40/aCTLA-4 therapy with aDEC-205/OVA vaccination rescued anergic tumor-specific CD8 T cells and significantly improved their activation, proliferation, and cytokine production (Fig. 1).

## Conclusions

Our recent studies suggest that finding appropriate vaccination methods to combine with checkpoint inhibitors (e.g., aCTLA-4) and costimulatory molecule agonism (e.g., aOX40) will be more effective at reducing tumor burden and improving survival than any single agent. In particular, the use of aOX40/aCTLA-4 alone was insufficient to eliminate larger, more established tumors, which may be due to increased Th2-associated cytokines or tumor-induced anergy [7]. One possible explanation for the reduced efficacy of combination therapy in the absence of vaccination is because it relies on TCR-mediated recognition of endogenous antigens. Due to mechanisms of central and peripheral tolerance the majority of these T cells are likely to be of low affinity for their respective tumor-associated antigens. In the absence of competition from T cells with higher affinity or an abundance of antigen, a Th2 response predominates [20, 21]. By administering both an adjuvant to promote DC maturation and a tumor-associated antigen targeted to an endocytic receptor present on DCs, we were able to very effectively prime an antitumor cytotoxic T lymphocyte (CTL) response. CTL activation through the TCR is known to induce expression of both OX40 and CTLA-4 receptors, thus providing targets for aOX40/aCTLA-4 therapy. This triple combination—using OX40 agonism to step on the gas, CTLA-4 blockade to release the brakes, and vaccination using aDEC-205/HER2 to steer the immune response in the right direction—was able to generate profound CTL infiltration into the tumor leading to tumor regression (Fig. 1). One possible explanation for the observed increase in Th1 polarization and concomitant reduction in Th2 cytokine production following triple combination therapy is that CTL-mediated cancer cell death will release an abundance of antigens, including those derived from over-expressed and/or mutated self-proteins. CD4 T cell recognition of these epitopes on mature antigen presenting cells expressing the appropriate costimulatory molecules would favor a Th1-polarized response. These and previous data also suggest an effect on CD8 T cell differentiation as a possible mechanism for the increased efficacy of the therapy. Our lab is currently investigating the molecular mechanisms underlying this process. Currently, there are multiple clinical trials testing various combinations of immune-based therapeutic modalities, including checkpoint inhibitors, targeted therapy with small molecule inhibitors, adoptive cell therapy, and standard of care chemotherapy or radiation. Dual treatment with CTLA-4 and PD-1 blockade (ipilimumab and nivolumab, respectively) was recently approved, and while it improves the overall response rate, the majority of patients succumb to their disease. The incidence of Grade 3-4 toxicities also increases with dual therapy, which is not surprising given the importance of these two molecules in preventing rampant autoimmunity. Perhaps combining a method of vaccination with a checkpoint inhibitor and a costimulatory molecule agonist, such as monoclonal antibodies activating OX40, 4-1BB, or GITR, will provide greater efficacy for patients, as it may more easily direct the immune response in the direction desired—away from normal self-antigens and toward a tumor-associated antigen. In the growing age of bioinformatics and personalized medicine, it seems that personalized vaccination is becoming a more feasible possibility for patients. Combining vaccination using a patient’s own tumor-associated neoepitopes with checkpoint inhibition and/or costimulatory molecule agonism will likely promote a more directed T cell response and may benefit a majority of patients, even with a minimal baseline presence of T cells. In fact, it is in this scenario where the efficacy of OX40 agonists may truly shine.

### Ethical approval and consent to participate

Not applicable.

### Consent for publication

Not applicable.

### Availability of supporting data

Not applicable.

## Abbreviations

CTL: cytotoxic T lymphocyte
DC: dendritic cell
TCR: T cell receptor
TIL: tumor infiltrating lymphocyte
